# Timing and cortical region matter: theta power differences between teenagers affected by Major Depression and healthy controls

**DOI:** 10.1007/s00702-024-02810-1

**Published:** 2024-08-06

**Authors:** Gideon Gradwohl, Sophia Snipes, Susanne Walitza, Reto Huber, Miriam Gerstenberg

**Affiliations:** 1 Lev Academic Center, Department of Computer Sciences, Jerusalem College of Technology, Jerusalem, Israel; 2 Child Development Center, University Children’s Hospital Zurich, University of Zurich, Zurich, Switzerland; 3 Children’s Research Center, University Children’s Hospital Zurich, University of Zurich, Zurich, Switzerland; 4 Neural Control of Movement Lab, Department of Health Sciences and Technology, ETH Zurich, Zurich, Switzerland; 5 Department of Child and Adolescent Psychiatry and Psychotherapy, Psychiatric University Hospital Zurich, University of Zurich, Zurich, Switzerland; 6 Neuroscicence Center Zurich, University of Zurich and Swiss Federal Institute of Technology Zurich, Zurich, Switzerland; 7 Zurich Center for Integrative Human Physiology, University of Zurich, Zurich, Switzerland; 8 Department of Child and Adolescent Psychiatry and Psychotherapy, Outpatient Services Winterthur, Psychiatric University Hospital Zurich, Albanistrasse 24, Winterthur, 8400 Switzerland

**Keywords:** Theta power, EEG, Major Depressive Disorder, Teenager

## Abstract

In adults affected by Major Depressive Disorder (MDD), most findings point to higher electroencephalographic (EEG) theta power during wake compared to healthy controls (HC) as a potential biomarker aiding the diagnostic process or subgrouping for stratified treatment. Besides these group differences, theta power is modulated by time of day, sleep/wake history, and age. Thus, we aimed at assessing if the time of recording alters theta power in teenagers affected by MDD or HC. Standardized wake EEG power was assessed with high-density EEG in 15 children and adolescents with MDD and in 15 age- and sex-matched HC in the evening and morning. Using a two-way ANOVA, group, time, and their interaction were tested. In patients, the current severity of depression was rated using the Children’s Depression Rating Scale. Broadband EEG power was lower in the morning after sleep, with a significant interaction (group x time) in central regions in the 4-6 Hz range. In MDD relative to HC, theta power was decreased over occipital areas in the evening and increased over frontal areas in the morning. A higher frontal theta power was correlated with more severe depressive mood in the morning but not in the evening. This was a cross-sectional study design, including patients on antidepressant medication. In conclusion, depending on time of recording, region-specific opposite differences of theta power were found between teenagers with MDD and HC. These findings stress the importance of the time of the recording when investigating theta power’s relationship to psychopathology.

**Supplementary Information:**

The online version contains supplementary material available at 10.1007/s00702-024-02810-1.

## Background

Earlier onset and longer duration of untreated depression is associated with worse outcome, higher suicidality, and more frequent recurrence (Kraus et al. 2019). This stresses the need for early identification and age-appropriate treatment approaches to reduce the burden of Major Depressive Disorder (MDD) and to ameliorate outcomes (Widge et al. 2019; Kraus et al. 2019). Thus, there have been high hopes for a biomarker of MDD in children and adolescents through the electroencephalogram (EEG), since it is non-invasive, cheap, and well tolerated by different age groups (Olbrich and Arns 2013; Gold et al. 2013; Widge et al. 2019; Newson and Thiagarajan 2019; de Aguiar Neto and Rosa 2019).

Theta spectral power (4–8 Hz) is one of the most studied waking oscillatory brain rhythms, altered in patients affected by MDD compared to healthy controls (HC) (Baskaran et al. 2012; Olbrich and Arns 2013; Arns et al. 2015; Wade and Iosifescu 2016; Pizzagalli et al. 2018). Its potential as a biomarker aiding early identification and the diagnostic process, or as a means for subgrouping patients for stratified effective treatment approaches, has been emphasized and critically discussed (Olbrich and Arns 2013; Gold et al. 2013; Wade and Iosifescu 2016; Pizzagalli et al. 2018; Newson and Thiagarajan 2019).

In a review, theta power was found to be increased frontally or globally across 18 studies in adult MDD patients compared to HC (Newson and Thiagarajan 2019). However, relative theta power, i.e. the ratio of theta power relative to other frequencies, did not differ between the groups, and other studies even reported opposite findings with overall lower theta power or region-specific occipital-parietal reductions in patients with MDD compared to HC (Fingelkurts et al. 2006; Mumtaz et al. 2017; Newson and Thiagarajan 2019; Lin et al. 2021).

The lack of standardization across studies, numerous options available for EEG analysis, different designs of the wake recording conditions and other methodological issues have been named as potential confounding factors contributing to diverse or even divergent results (Olbrich and Arns 2013; Poldrack et al. 2017; Widge et al. 2019).

Independently from psychopathology, theta power is also affected by prior sleep-wake history and the homeostatic regulation of sleep. Theta power increases with time spent awake and decreases following sleep, thus reflecting sleep pressure, with additional fluctuations according to circadian rhythms (Aeschbach et al. 1997; Dumont et al. 1999; Finelli et al. 2000). Theta power changes due to sleep pressure are highest in frontal areas and further depend on the quality of prior wake activity as well as ongoing mental activity during the EEG measurement (Hung et al. 2013; Bernardi et al. 2015; Snipes et al. 2022). Altogether, these results indicate that sleep pressure, and therefore time of day, can impact theta power in both global and region-specific ways.

The interaction between these homeostatic changes in theta power and those related to MDD has not been explored. This is potentially problematic as previous studies rarely report a specific time window during which the wake EEG was performed, nor control for prior sleep quality and duration, adding to the lack of standardization within study protocols especially when comparing patient groups and controls (Mumtaz et al. 2017; Widge et al. 2019). Further, the potential impact of developmental aspects has rarely been addressed. Even though depression often emerges during late childhood and adolescence, and EEG power undergoes significant age-related changes during development, only few studies have carefully assessed theta power in this age-group (Carskadon et al. 2004; Jenni et al. 2005; Campbell and Feinberg 2009; Hagenauer et al. 2009). One study in affected children and adolescents reported lower theta power in the occipital-parietal regions compared with HC (McVoy et al. 2019a). The clinical presentation of MDD is often heterogeneous, especially during childhood and adolescence, and a biomarker would be particularly supportive during the diagnostic process for this young age group to identify the disorder early and initiate targeted treatment (Steiger and Kimura 2010; McVoy et al. 2019b; Zwolińska et al. 2023).

Therefore, we aimed to assess group- and time-specific alterations in a broad frequency range (2–12 Hz) spanning the canonical theta band (4–8 Hz) using high-density EEG wake data at two specific time points, the morning and evening, in teenagers affected by MDD or HC. Regarding group-specific differences, we expected higher levels of theta power in teenagers with MDD compared to HC based on the majority of findings in adults. Regarding time-specific aspects, we expected both groups to show higher theta in the evening compared to the morning. We further investigated whether there was an interaction between group and time of recording. Finally, we explored the associations of theta power with current severity of depression in patients.

## Methods

### Participants

All participants met the following criteria: (1) aged 12–18, (2) IQ > 70, (2) no major medical or neurological conditions known to affect the brain, including history of significant head injury, (3) no substance use dependence within the past 6 months, (4) no diagnosed sleep disorder.

HC additionally had to have (1) no diagnosis of a mental disorder and (2) no use of medication.

The initial 19 patients which met the criteria for MDD first single episode or recurrent according to DSM-IV were recruited from in- and outpatient settings at the Department of Child and Adolescent Psychiatry and Psychotherapy of the Psychiatric University Hospital of Zurich, Switzerland (American Psychiatric Association 2000; Gerstenberg et al. 2020). DSM-IV Axis I diagnoses were confirmed using the Mini International Neuropsychiatric Interview for Children and Adolescents (MINI-KID) (Sheehan et al. 1998, 2010). In this study, the final sample comprised 15 patients with a full set of wake EEG data at comparable time points in the evening and in the morning (Table 1). Sleep data from this cohort was previously published (Tesler et al. 2016; Gerstenberg et al. 2020).

**Table 1:** Demographic characteristics, wake recording and sleep structure information of patients with Major Depressive Disorder and healthy controls

	MDD (*n* = 15)	HC (*n* = 15)	*p*-value
Demographic characteristics			
Age, years (mean ± SD)	15.17 ± 1.10	15.20 ± 1.17	0.90
Sex, n females (%)	10 (66.7%)	10 (66.7%)	
**Wake recordings** (mean ± SD)			
Time of evening wake recording	21:44 ± 0:47	21:39 ± 0:29	0.84
Bedtime	22:31 ± 0:41	22:47 ± 0:27	0.36
Wake-up time	6:34 ± 0:31^a^	7:19 ± 1:11	**0.03**
Time of morning wake recording	6:51 ± 0:36	7:37 ± 1:16	0.06
Duration between wake up and time of morning wake recording (min)	15.52 ± 7.87^a^	18.17 ± 7.54	0.23
**Sleep structure** (mean ± SD)			
Sleep onset latency (min)	20.81 ± 14.91	21.62 ± 13.53	0.66
REMS latency (min)	108.69 ± 31.41	125.93 ± 48.80	0.50
Wake after sleep onset (min)	10.76 ± 6.82	33.50 ± 32.30	**0.00**
Sleep stage 1 (%)	5.91 ± 3.08	5.48 ± 1.68	0.66
Sleep stage 2 (%)	48.20 ± 7.06	47.69 ± 5.38	0.77
Sleep stage 3 (%)	19.49 ± 4.92	18.44 ± 4.93	0.60
REMS (%)	20.29 ± 5.07	17.65 ± 4.39	0.11
Total sleep time (min)	447.05 ± 50.45	450.71 ± 82.41	0.77
Total time in bed (min)	475.57 ± 43.66	503.12 ± 72.46	0.32
Sleep efficiency (%)	93.90 ± 4.39	89.25 ± 7.42	0.06

HC = healthy controls, MDD = Major Depressive Disorder, REMS = Rapid Eye Movement Sleep, SD = standard deviation. Non-Rapid Eye Movement Sleep stages 1, 2, 3 and Rapid Eye Movement Sleep (REMS) are presented as percentage of ‘total time in bed’. ‘total sleep time’ includes stages 1, 2, 3 and REMS and ‘sleep efficiency’ is calculated as the percentage of ‘total sleep time’ of the ‘total time in bed’. ^a^*n*=14 because wake up time was not available for one patient. Bold values represent significant group differences. All values were compared with Mann-Whitney U-tests

Stable treatment with psychotropic medication was not an exclusion criterion in MDD patients. In total, nine out of 15 patients (60.0%) received psychotropic medication. Eight patients (53.3%) received selective-serotonine receptor inhibitors: fluoxetine (*n* = 4; 26.7%), sertraline (*n* = 3; 20.0%), citalopram (*n* = 1; 6.7%). For those individuals, a fluoxtine equivalent was derived (Hayasaka et al. 2015). The current severity of depression was assessed using the total score of the Children’s Depression Rating Scale Revised (CDRS) (Poznanski et al. 1985; Keller et al. 2011). The mean of the total score was 42.33 ± 10.43. Further, five subscores (observed depressive mood; anhedonia; morbid thoughts; somatic symptoms; reported depressive mood) were derived (Guo et al. 2006).

The Wechsler Intelligence Scale for Children IV was used in all patients to assess overall cognitive performance (Laney et al. 2011). It was performed in a stable phase of disease within one year of the EEG recordings in all patients. The full version or a short version was performed within two weeks of the EEG recordings in HC (Waldmann 2008).

HC underwent a telephone and questionnaire screening to exclude personal and family history of mental disorders, and use of medication. Sleep data from HC was collected in other studies using the same experiment protocol as for the MDD patients, matched by age and sex.

### Wake and sleep EEG signal recording

Wake EEG and sleep assessment were conducted under identical conditions in both groups. During the four minutes of wake EEG in the evening and in the morning, participants were engaged in an auditory oddball task following Fattinger et al. (2017). Briefly, participants were instructed to sit quietly in front of a screen and fixate on a cross. Tones were played every 0.8 s, and a random 10% of the 300 tones were deviants to which participants had to press a mouse button in response as fast as possible. This simple task was chosen instead of classic resting EEG because it standardized the rather long duration of wake EEG for teenagers who may otherwise struggle to sit still for so long, and to prevent them from falling asleep. Previously, we showed that changes in theta activity with sleep pressure are comparable between resting and oddball EEG (Snipes et al. 2023).

All participants were requested not to consume alcohol or caffeine 24 h before the experiment, to keep their regular sleep-wake cycles for at least 7 days before the recording, and not to take naps during the daytime. Self-reported sleep-wake logs and wrist motor actigraphy (Actiwatch Plus, AW4, Cambridge Neurotechnology, Cambridge, England) validated the compliance to the instructions. Sleep data were visually scored in 20 s epochs according to the American Academy of Sleep Medicine standard criteria (Iber and Iber 2007).

The EEG signal was measured with a high-density cap of 128 channels (HydroCel Geodesic Sensor Nets™), amplified (EGI Amplifiers) and recorded (EGI NetStation software) with a sampling frequency of 1000 Hz, and Cz reference. We restricted our analyses to the 112 electrodes above the mastoids, excluding all face, neck, and ear electrodes. The data was filtered in a frequency band of 0.5–50 Hz and downsampled to 250 Hz. Data preprocessing, analysis, and statistics were done with custom scripts in MATLAB (R2019b) based on the EEGLAB toolbox v2019.1 (Delorme and Makeig 2004). Specifically, preprocessing war performed according to the procedure described by Snipes et al. (2022). Briefly, artefact channels and time points were removed manually. Then, independent component analysis (ICA) was used to separate physiological artefacts such as eye blinks, saccades, muscle tone, and heartbeat. These artifacts were then removed with a semi-automatic procedure.

### Statistical analysis

Demographic characteristics, wake recording and sleep structure information of patients with MDD and HC were compared with Mann-Whitney U-tests to account for non-normally distributed data.

EEG power spectral density was computed with MATLAB’s ‘pwelch’ function with 4 s Hanning windows and 50% overlap. EEG power at each frequency was then z-scored for each participant, pooling across channels and timepoints. This was done to reduce the variance in power due to inter-individual differences, as done in Snipes et al. (2022). Z-scoring in this way increases sensitivity to local differences in power. The trade-off is that any group-level differences in the average power would be lost. We confirmed that no such difference existed in the untransformed data (Supplementary Material 1).

**Fig. 1:**
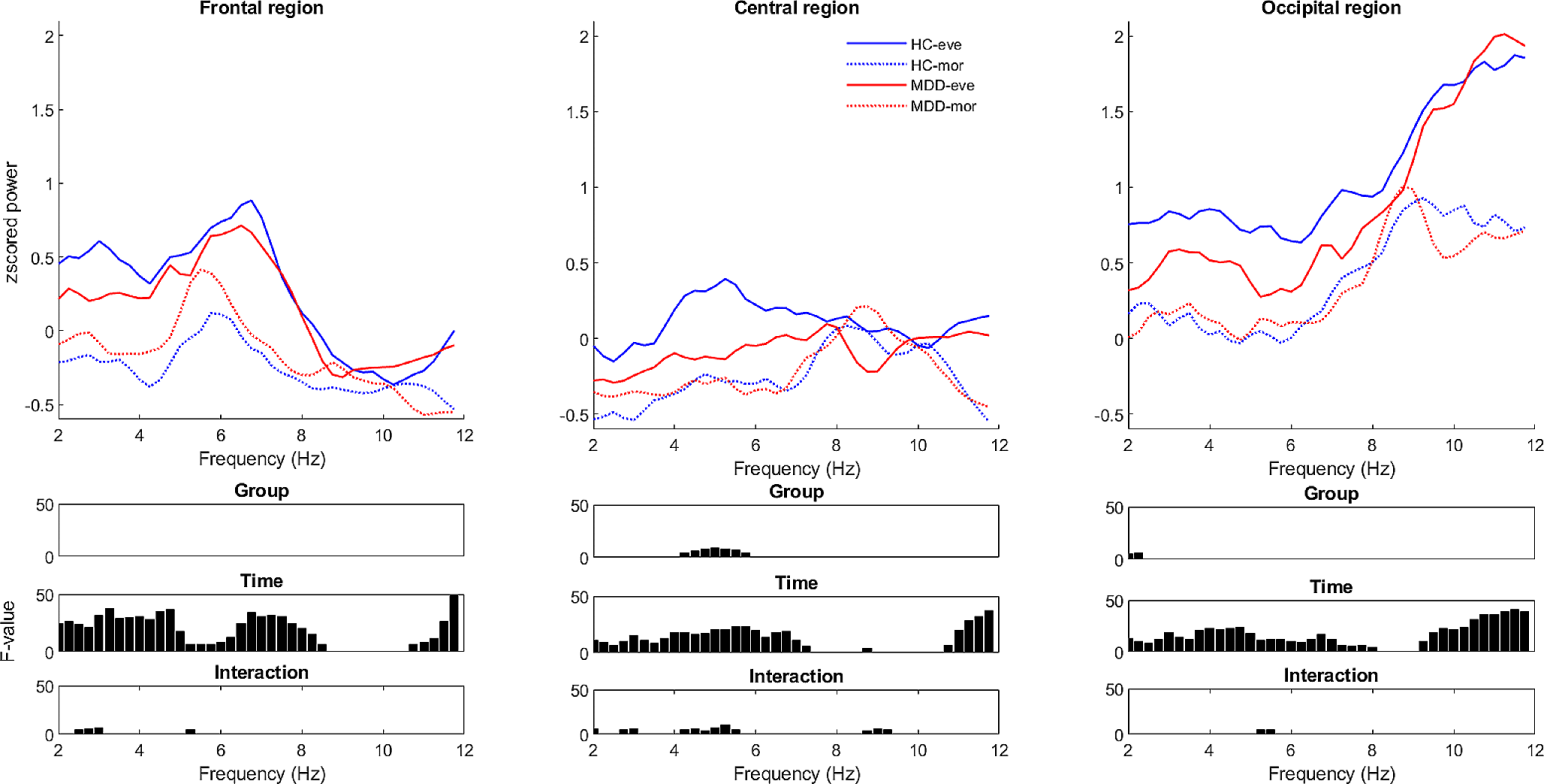
Z-scored power spectra during wakefulness in the evening and the morning of patients with MDD and HC across 3 regions of interest. First row illustrates mean z-scored EEG power spectra during wake recordings of patients with Major Depressive Disorder (MDD, *n* = 15, red lines) and healthy control participants (HC, *n* = 15, blue lines) at the frontal, central and occipital regions, measured in the evening (-eve; solid lines) and morning (-mor; dotted lines). The following rows represent FDR corrected F-values of 2-way ANOVA factors ‘group’, ‘time’ and their interaction

Z-scored EEG wake data was statistically evaluated using two-way ANOVAs with factors group (MDD, HC), time (mor, eve) and their interaction for each frequency bin (Fig. 1). To reduce the number of tests, we focused on three ‘Regions Of Interests’ (ROI). The selection of the electrodes belonging to either the frontal, central, or occipital region was the same as in Snipes et al. (2022). To account for multiple comparisons, we used false discovery rate (FDR) correction (Benjamini and Hochberg 1995).

For the topographical distribution and comparison, z-scored theta power values were averaged between 4 and 8 Hz for each electrode. Statistical comparisons at each electrode were assessed by Student’s t-test, paired for overnight comparisons and unpaired for between-group comparisons. To account for multiple comparisons in these tests, we applied statistical nonparametric mapping (SnPM) to determine the critical cluster size (Nichols and Holmes 2002).

To assess associations of theta power with severity of depression, untransformed theta power was averaged into the same ROIs, and correlated to the Children’s Depression Rating Scale (CDRS) total score and five subscores using Pearson’s correlations.

## Results

First, sleep architecture and schedule differences were compared between MDD and HC to ensure comparable sleep conditions (Table 1). The timepoints of evening and morning wake recordings did not significantly differ between the groups. Only the wake-up time in the morning was significantly later in HC (*p* = 0.03).

None of the sleep architecture parameters were statistically different between MDD and HC, except for wake after sleep onset, which was on average 11 min for MDD, and 34 min for HC (*p* < 0.00). This resulted in a trending reduction in sleep efficiency for HC, although in both groups, it was sufficiently high to be considered good sleep quality (Ohayon et al. 2004). Altogether, this indicates that the sleep between evening and morning recordings was comparable between groups, and if anything, HC slept a little worse.

To determine the specificity of any EEG spectral changes to the theta range, we first investigated the changes in the wake EEG power spectrum including the frequency ranges adjoining the theta range in both directions (2–12 Hz) during the evening and morning session for both groups in three ROIs (Fig. 1, z-scored within each frequency bin; for untransformed values, see Supplementary Material 1). Power significantly decreased following sleep for all frequencies except 9–11 Hz in the frontal ROI, 7.5–11 Hz in the central ROI, and 8–9 Hz in the occipital ROI.

We found significant main group effects between 4 and 8 Hz in the center ROI. In addition, within the same frequency range a significant interaction between group and time was observed. This interaction was largely driven by a prominent peak in the theta range in the evening of HC participants, which was not present in MDD participants, and was not present for both groups in the morning. Significant interactions were also found in the delta range of the frontal ROI (~ 3 Hz), and the lower-alpha range (~ 9 Hz) of the central ROI.

Altogether, we found that sleep has broadband time of day effects on wake EEG power for both MDD and HC, and there are significant interactions with group in multiple bands, with the largest being in the theta range for the central ROI.

**Fig. 2:**
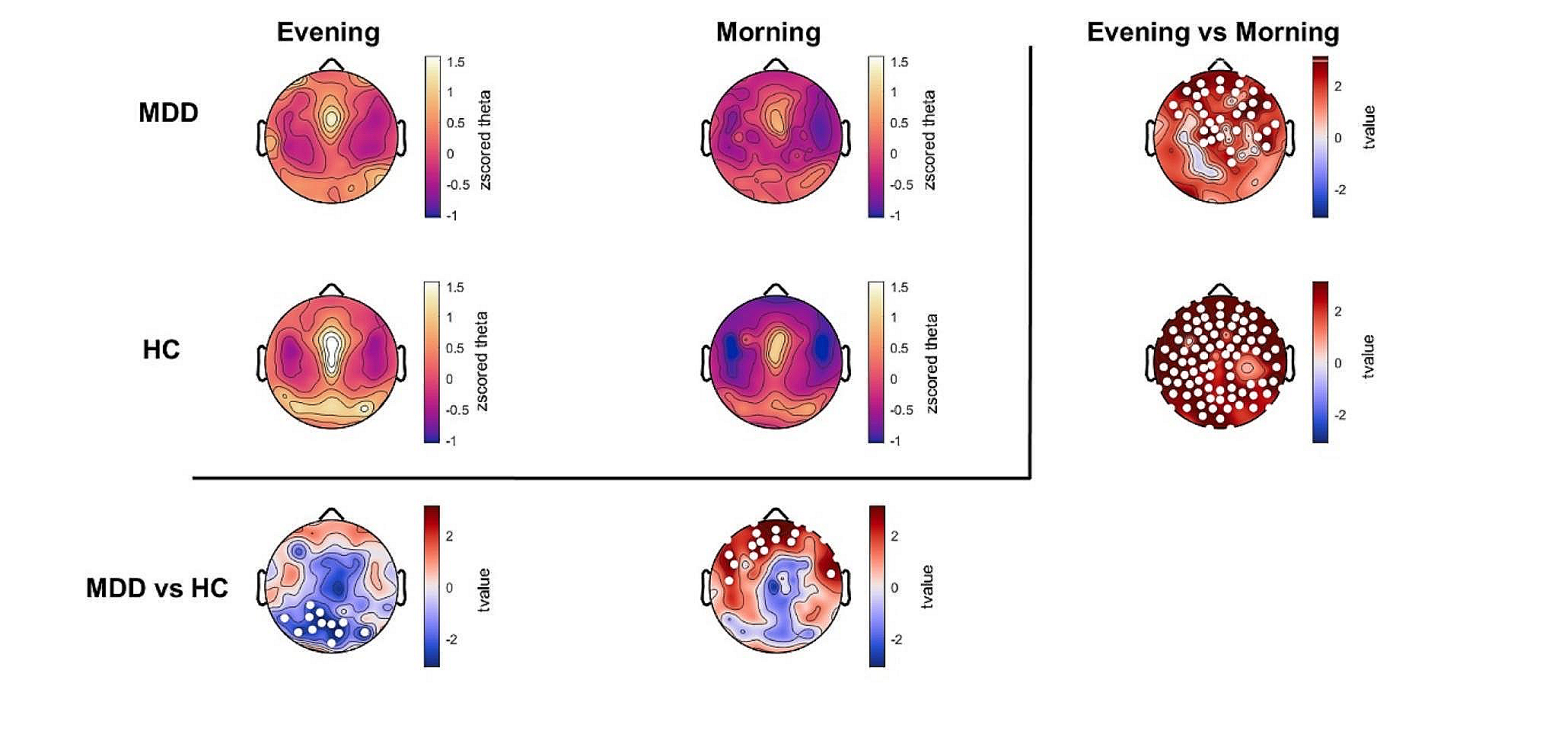
Topographical distribution of EEG theta power during wakefulness in MDD and HC groups in the evening and in the morning. Purple to yellow topographies indicate average z-scored theta power (4–8 Hz) for each group at each timepoint. Red to blue topographies in the lowest row indicate t-values from t-test comparisons between MDD and HC at each timepoint, such that red indicates greater theta in MDD patients. Red/blue topographies in the right-most column indicate within-subjects paired t-test comparisons from evening to morning, such that red indicates greater theta in the evening. White dots indicate statistically significant channels, cluster corrected for multiple comparisons

To further investigate the power changes in the 4–8 Hz frequency range we calculated theta power for each electrode, in the evening and morning for both groups (Fig. 2). Both timepoints for both groups showed the typical topographical distribution of theta power, with maxima over fronto-central and occipital regions (Fig. 2).

Theta power was significantly higher in the evening compared to morning in 93% of all electrodes in HC participants and 33% of all electrodes in MDD, clustering over the fronto-central region (Fig. 2, third column). Comparing MDD versus HC participants separately in the evening and morning revealed region-specific differences: In the evening, MDD patients showed significantly lower theta power than HC over 16% of all electrodes, clustering over the occipital region (Fig. 2, third row, left plot). In the morning, MDD patients showed higher theta values over 19% of all electrodes compared to HC, clustering over the frontal region (Fig. 2, third row, right plot).

**Fig. 3:**
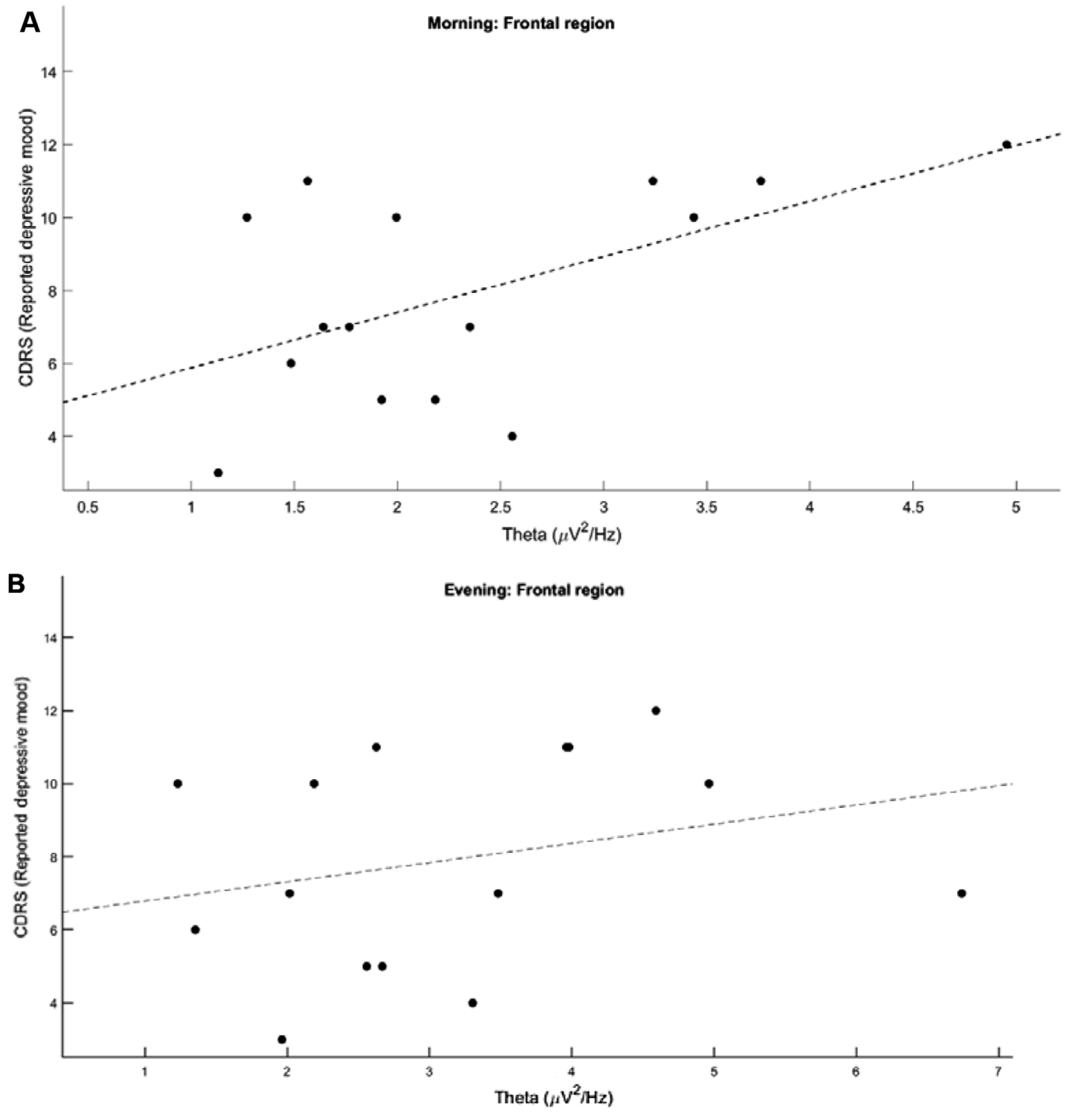
Correlations between theta power and reported depressive mood according to the Children’s Depression Rating Scale in the morning (**A**) and evening (**B**)

Finally, to investigate the relationship between theta power and actual symptom severity, explorative Pearson’s correlations were performed within MDD patients. The Children’s Depression Rating Scale (CDRS) total score and five subscores were correlated with theta power at each ROI in the evening and morning. We observed a significant positive correlation between morning theta power and the CDRS subscore ‘reported depressive mood’ (*r = 0.55*, *p = 0.03*) over the frontal ROI (Fig. 3A), although this finding did not survive Bonferroni correction for multiple testing (corrected p-value *p* = 0.15). The relationship for the evening EEG was in the same direction but did not reach significance even without correction for multiple comparisons (Fig. 3B, r *= 0.27*, *p = 0.34*). There were no other significant correlations between theta power and overall severity of depression, or any of the other four subscores (observed mood, anhedonia, morbid thoughts, somatic symptoms), at any other ROI. To control whether the effect could be driven by medication, we also correlated theta power at each ROI and each timepoint with the fluoxetine equivalent, and found no significant relationship (*p* > 0.056).

## Discussion

In teenagers with MDD compared to HC, depending on time of the day, we found region-specific opposite differences in theta power; specifically higher frontal theta power in the morning and lower occipital theta power in the evening. Thus, our findings are both in agreement and in disagreement with existing literature.

In agreement with the majority of findings in adult patients affected by MDD, teenagers with MDD showed higher theta power over frontal areas compared to HC in the morning (Newson and Thiagarajan 2019). Most likely, these studies in adults were conducted during normal working hours, closer to the morning than late evening, although the actual measurement times are not reported.

There are few studies in patients with an affective disorder reporting multiple recordings during the day in the context of extended wakefulness protocols (Cajochen et al. 2000; Danilenko and Putilov 2005; Birchler-Pedross et al. 2011). In one study, theta power build-up was assessed in 16 female patients affected by depression with a seasonal pattern of recurrence, compared to HC (Danilenko and Putilov 2005). In this study, theta power values of the fronto-central deviation were reported for 5 time points during the day before the sleep deprivation protocol. Based on visual inspection of the curve over the course of the day, theta appears higher in patients with MDD in the morning (10 am), at 1pm there is a convergence, in the afternoon (4 pm) theta power appears higher in HC, and during the two evening measurements (7 and 10 pm) there are identical power values. Considering absolute theta power, the authors did not find statistical differences between the groups at the respective time points, but theta power relative to the mean was significantly higher in the morning in patients with MDD (Danilenko and Putilov 2005). This corresponds to our finding of higher frontal theta power in the morning and no differences in frontal areas in the evening. A comparison with two further studies reporting multiple daytime recordings in patients is hampered because one study focused on a frequency band below the theta range and another study included only 8 female patients and a broad frequency band overlapping with the alpha frequency band (Cajochen et al. 2000; Birchler-Pedross et al. 2011). This combination of frequency ranges is problematic because whereas theta power increases, alpha power decreases during the day in adults (Aeschbach et al. 1997; Strijkstra et al. 2003; Snipes et al. 2023).

In disagreement with the above-mentioned findings, teenagers with MDD showed lower theta power over occipital areas compared to HC in the evening. Comparing this finding to existing literature is hampered due to the lack of previous studies reporting the specific time windows for EEG recordings and/or region-specific effects. However, it seems in line with one study reporting theta power to be reduced particularly in parietal and occipital regions in adolescent patients affected by MDD (McVoy et al. 2019a). Further, two studies in adult patients affected by MDD reported lower theta power globally across frontal, temporal, central, parietal and occipital regions compared to healthy controls (Mumtaz et al. 2017; Lin et al. 2021).

Given the several factors affecting theta power in opposing directions, interpreting the meaning of region and time dependent theta power changes is challenging.

On the one hand, local theta power increases seem to be driven by increased daytime use of specific cortical areas (Hung et al. 2013; Bernardi et al. 2015). Specifically, the more experience-dependent neural plasticity occurs in a given network, the more theta power increases. MDD patients differ significantly from healthy controls both from a behavioural and a neural plasticity perspective, which would be expected to result in region and time dependent differences in theta power compared to healthy controls (Liu et al. 2017; Appelbaum et al. 2023).

On the other hand, theta power increases also seem due to an “idling mode” of cognitive control networks, i.e. when a cortical region is not in use, theta power locally increases in that area (Scheeringa et al. 2008, 2009; Michels et al. 2010; Snipes et al. 2022). Vice-versa, when engaging with a specific task or mental activity, areas involved in this task show reduced theta power. This anti-correlation between theta power and local activity was specifically observed in frontal midline areas, and such areas are involved in ruminative thinking typically seen in MDD patients (Scheeringa et al. 2008; Drevets et al. 2008; Burkhouse et al. 2017; Kaiser et al. 2019; Rosenbaum et al. 2021). Therefore, it is possible that such idling theta could differ between patients and controls, and could even differ between recording times.

These opposing forces of theta power changes, i.e. use-dependent or idling-related, may mask each other and thus prevent a conclusive interpretation of the time and region dependent changes in theta power seen in MDD patients in our study. For future studies, one option would be to control for mental activity by recording the EEG during an engaging task that will involve the same brain networks in patients and HC.

There could also be fundamental differences in how theta oscillations are generated in patients, which could either be an epiphenomenon (e.g. reflecting structural differences), or altered theta may actually contribute to the pathology itself. Such a difference would not in theory change with time of day or recording type, but it may be more or less apparent depending on such conditions. In all likelihood, many if not all of these factors will combine at different times of day, resulting in varied theta topographies, thus complicating any potential interpretation. This would also explain why correlations between theta power and symptom severity are stronger in the morning than in the evening. It could well be that theta in the morning more specifically reflects specific core symptoms of pathology, i.e. the subscore of more severe “reported depressive mood” comprising self-reported depressive symptoms such as low self-esteem, depressive mood and frequent crying (Disner et al. 2011). This association may then be masked by sleep-wake or time of day factors affecting theta activity. Therefore, it may still be possible to find a practical application for a readout like theta power when properly controlling for variables such as ongoing mental activity and time of day.

Finally, the impact of maturational aspects on theta power cannot be disentangled based on the current findings. It is possible that developmental differences in theta power in teenagers affected by MDD were more likely to have acted via homeostatic or circadian components (Carskadon et al. 2004; Crowley et al. 2007; Blake et al. 2018). Still, a lack of studies assessing wake theta power longitudinally in young patients with MDD or studies with a direct comparison of children and adult patient groups prevent further conclusions.

Another finding in this study was that all individuals, irrespective of their health state, showed lower levels of EEG power in a broad frequency band in the morning compared to the evening, excluding the alpha band. After a comparable night with high sleep efficiency in both groups, this overall time-dependent difference could be found in all three regions across the scalp. One previous study in adults also reported an overnight decline of broad frequency activity (3.25–6.25 Hz, 11.75–21.5 Hz) in HC Plante et al. 2013. Further, they found the same direction of the change but no statistically significant effect in a group of young adults affected by MDD (Plante et al. 2013). It seems that healthy teenagers or adults show stronger overnight reductions than patients affected by MDD. This is in line with the topography results in teenagers of this study, showing widespread effects across the whole scalp in HC, but only over frontal areas in patients affected with MDD. Thus, an altered overnight decline as well as an altered build-up of low frequency oscillations during the day may have influenced theta power globally or with a region-specific predominance in both adults and teenagers affected by MDD compared to HC.

### Limitations

These findings must be viewed in the context of several limitations. The moderate n-size and different underlying neuropathomechanisms of the depressive episode in teenagers may have influenced the results. MDD in its cross-sectional symptom presentation as well as in its longitudinal course is a heterogeneous disorder. Especially during first onset and during childhood and adolescence, future seasonal patterns of recurrence or transition to a bipolar disorder cannot be determined and therefore not be controlled for in this cross-sectional study design (Rao et al. 2002; Akiskal 2005). Further, medication was not an exclusion criterion and may have influenced EEG power. To address this limitation, a fluoxetine equivalent was calculated and there were no associations of the reported group-differences with medication intake. Additionally, daytime activity and engagement in demanding cognitive tasks were not controlled for in this study but may be confounding factors contributing to local increases of theta power what might be more relevant in the evening and may differ between the groups (Jensen and Tesche 2002; Hung et al. 2013; Bernardi et al. 2015; Murphy et al. 2019).

Additionally, the wake recording was assessed using an oddball paradigm with a substantial number of repetitive acoustic stimuli and 10% of oddball trials. It cannot be ruled out that there may be group-related differences especially regarding the oddball trial. Still, this refers to a very small proportion of the total wake EEG data and it is highly unlikely to have driven the overall group differences which are contrasting in direction and region- and time specific. Furthermore, it was not possible with this data to disentangle circadian from homeostatic effects. Considering circadian effects, the so-called “wake maintenance” or “forbidden zone” may have influenced the recordings in the evening. The wake maintenance zone is a 2–4 h window before habitual sleep onset when theta power drops drastically and individuals find it difficult to initiate sleep (Cajochen et al. 2002; Zeeuw et al. 2018; Snipes et al. 2023). However, little is known about the wake maintenance zone in teenagers or in patients with MDD. It cannot be excluded that despite a similar time point of the evening recording in both groups, young depressed individuals may be more susceptible to or stay longer in the wake maintenance zone. On the other hand, regarding the morning recordings, sleep inertia may have interacted with spectral power (Hilditch and McHill 2019). Finally, it is important to note that these results came from data z-scored for each participant; therefore, for each frequency, any group-level differences between MDD and HC in power stable across channels and timepoints were lost.

## Conclusions

To conclude, the time of the recording matters when assessing theta power in 12–18 year-olds with MDD and relating potential group-differences to psychopathology. Likewise, the opposite region-specific differences also add to the critical discussion of the potential of theta power as a biomarker for early detection of depression, especially in a group of young patients with MDD.

### Electronic supplementary material

Below is the link to the electronic supplementary material.

Supplementary Material 1
