# Supplementary material for: Timing and cortical region matter: theta power differences between teenagers affected by Major Depression and healthy controls
Source: J Neural Transm (Vienna). 2024 Aug 6;131(9):1105–15. doi: 10.1007/s00702-024-02810-1 (PMC11365826; doi:10.1007/s00702-024-02810-1)
Supplement: Supplementary file 1 — Supplementary Material 1 [file 702_2024_2810_MOESM1_ESM.docx]

**Supplementary Material 1:**

**Fig. 4**


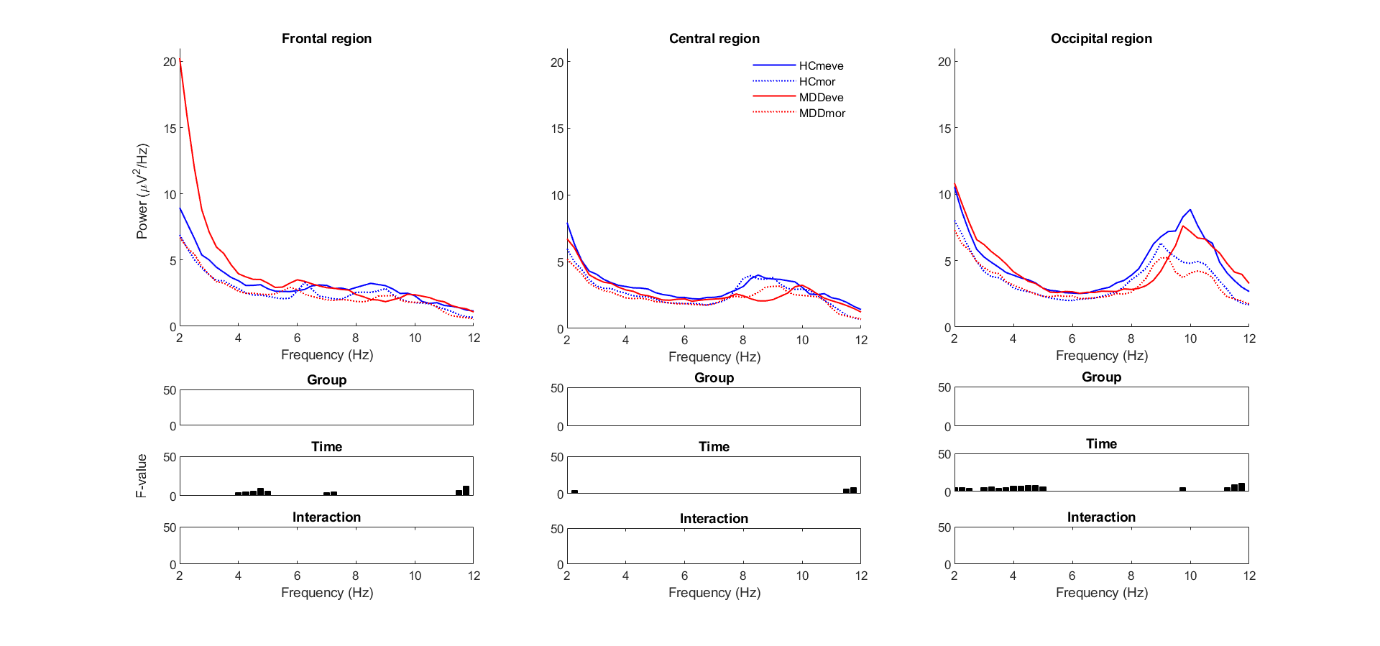


Power spectra during wakefulness in the evening and the morning of patients with Major Depressive Disorder (MDD) and healthy controls (HC) across the frequency range 2-12 Hz and 3 regions of interest. First row illustrates mean EEG power spectra during resting wake recordings of patients with MDD (n=15, red lines) and HC (n=15, blue lines) at the frontal, central and occipital regions, measured in the evening (solid lines) and morning (dotted lines). The following rows represent FDR corrected F-values of 2-way ANOVA factors ‘group’, ‘time’ and their ‘interaction’.
